# Gut microbiota-bile acid-immune axis: receptor signaling, metabolic regulation, and therapeutic translation

**DOI:** 10.3389/fimmu.2026.1910940

**Published:** 2026-10-09

**Authors:** Ruixin He, Qiangqiang Zheng, Yi Shen

**Affiliations:** 1 Anesthesia & Operation Center, West China School of Public Health and West China Fourth Hospital, Sichuan University, Chengdu, Sichuan, China; 2 Department of Thoracic Surgery, West China School of Public Health and West China Fourth Hospital, Sichuan University, Chengdu, Sichuan, China

**Keywords:** bile acids, gut microbiota, immune regulation, metabolic control, receptor signaling, therapeutic translation

## Abstract

The gut microbiota and bile acid metabolism are intricately linked, forming a pivotal axis that regulates host immune function and metabolic homeostasis. Bile acids not only facilitate lipid digestion as surfactants but also act as key signaling molecules by activating multiple receptors, which modulate immune responses, energy metabolism, and inflammatory processes. The gut microbiota enzymatically converts primary bile acids into secondary bile acids, thereby influencing the composition and function of the bile acid pool. Dysbiosis of the gut microbiota and aberrations in bile acid signaling pathways are closely associated with various diseases. Recently, therapeutic strategies targeting the gut microbiota-bile acid-immune axis have emerged, including receptor agonists/antagonists and microbiota modulators, offering novel approaches for disease prevention and treatment. This review comprehensively summarizes the molecular mechanisms, receptor signaling pathways, metabolic regulation, and clinical translational potential of this axis, aiming to provide theoretical support for future precision medicine and innovative therapeutic strategies.

## 1 Introduction

The interplay between the gut microbiota, bile acid metabolism, and host immunity constitutes a fundamental axis for physiological homeostasis. Gut microbes enzymatically convert hepatic primary bile acids into diverse secondary derivatives, which in turn reshape the microbial community itself. This bidirectional crosstalk regulates intestinal barrier integrity, immune cell differentiation, and systemic metabolism, thereby influencing host health and disease susceptibility (1–3). Beyond their classical role in lipid digestion, bile acids act as signaling molecules that engage nuclear receptors—farnesoid X receptor (FXR), pregnane X receptor (PXR), and vitamin D receptor (VDR)—and membrane receptors such as G protein-coupled bile acid receptor 1 (GPBAR1/TGR5) and sphingosine-1-phosphate receptor 2 (S1PR2) to regulate inflammation, energy metabolism, and intestinal homeostasis (2, 4, 5).

Microbial deconjugation, dehydroxylation, and epimerization generate secondary bile acids such as deoxycholic acid (DCA) and lithocholic acid (LCA), which possess distinct immunomodulatory properties (3, 6). These metabolites shape the differentiation and function of macrophages, dendritic cells, regulatory T cells, and T helper subsets, thereby sustaining immune tolerance and limiting excessive inflammation (1, 7). Dysbiosis and altered bile acid profiles have accordingly been implicated in inflammatory bowel disease (IBD), primary biliary cholangitis (PBC), metabolic syndrome, liver fibrosis, and neurodegenerative disorders (8–11).

In IBD, depletion of bile acid-transforming bacteria causes primary bile acids to accumulate at the expense of secondary bile acids, impairing receptor signaling and barrier function and aggravating mucosal inflammation (8, 12). In cholestatic liver diseases such as PBC and primary sclerosing cholangitis (PSC), dysbiosis and bile acid imbalance promote immune-mediated bile duct injury through aberrant receptor and immune cell activation, underscoring the role of the gut-liver axis in disease progression (9, 13). Obesity, type 2 diabetes, and dyslipidemia are likewise linked to microbiota and bile acid alterations that modulate systemic inflammation through receptor-mediated signaling (10, 14).

These insights have motivated therapeutic strategies targeting the axis, including bile acid receptor agonists and antagonists, probiotics, prebiotics, fecal microbiota transplantation (FMT), and dietary interventions, all of which show potential to ameliorate immune and metabolic dysregulation (2, 8, 15). Defining how bile acid metabolites shape immune cell metabolism further opens avenues for immunomodulatory therapy in autoimmune, inflammatory, and metabolic disease (16, 17). Notably, the axis also reaches extraintestinal organs and the central nervous system through the gut-brain axis, broadening its physiological and pathological relevance (18).

This review therefore summarizes current understanding of receptor-mediated signaling, metabolic regulation, and translational therapeutic strategies centered on the gut microbiota-bile acid-immune axis, with emphasis on how disruption of this hub contributes to disease and how its components may be targeted clinically.

## 2 Regulatory mechanisms of gut microbiota on bile acid metabolism

### 2.1 Primary and secondary bile acid biosynthesis and transformation

Primary bile acids, chiefly cholic acid (CA) and chenodeoxycholic acid (CDCA), are synthesized in the liver from cholesterol by cytochrome P450 enzymes including CYP7A1, CYP27A1, and CYP8B1. They are conjugated with glycine or taurine to increase solubility, secreted into bile, and released into the intestinal lumen to facilitate fat digestion and absorption, with minor synthesis reported in extrahepatic tissues such as the ovaries (19). Within the intestine, primary bile acids undergo extensive microbial transformation that profoundly reshapes the bile acid pool and, consequently, host metabolism and immunity.

The gut microbiota converts primary into secondary bile acids through deconjugation, dehydroxylation, oxidation, and epimerization. The initial and rate-limiting step is deconjugation by microbial bile salt hydrolases (BSHs), which hydrolyze the amide bond linking bile acids to glycine or taurine and thereby enable all subsequent modifications. BSH activity is widespread among gut bacteria, including *Bacteroides*, *Clostridium*, *Lactobacillus*, and *Bifidobacterium* (20–22). Deconjugation alters bile acid solubility and toxicity and modulates host FXR and TGR5 signaling, thus influencing metabolic and immune homeostasis (23, 24).

Specific taxa subsequently perform 7α-dehydroxylation, converting primary bile acids into deoxycholic acid (DCA) and lithocholic acid (LCA). This reaction is catalyzed by enzymes encoded in the bile acid inducible (bai) operon, predominantly in *Clostridium* species (21, 25). The abundance and activity of these bacteria directly determine the secondary bile acid pool, which modulates intestinal inflammation, carcinogenesis, and metabolic disease (19, 26); increased secondary bile acid biosynthesis has, for example, been linked to colonic inflammation through LCA and its interaction with immune signaling pathways (26).

Other microbial transformations include oxidation and epimerization of hydroxyl groups on bile acids, leading to the formation of iso- and epi-bile acids with distinct biological activities. Enzymes such as hydroxysteroid dehydrogenases (HSDHs) mediate these reactions, and their genes have been identified in various gut bacteria, including *Bacteroides* and *Collinsella* species (25, 27). These modifications further diversify the bile acid pool and influence host receptor activation.

The composition of the gut microbiota and its enzymatic repertoire are critical determinants of bile acid metabolism. Dysbiosis, whether due to disease, antibiotic use, or dietary changes, can alter bile acid profiles, leading to metabolic disturbances and disease progression. For example, antibiotic-induced microbiota disruption reduces BSH activity and secondary bile acid production, impairing bile acid signaling and host metabolism (19, 28). Conversely, probiotic supplementation with BSH-producing bacteria such as *Lactobacillus gasseri* can enhance primary bile acid biosynthesis and promote secondary bile acid transformation, benefiting the gut-liver axis (29).

Moreover, bile acid metabolism by the gut microbiota exhibits circadian rhythmicity and is influenced by host factors such as diet, age, and disease state. Advanced techniques including metagenomics, metabolomics, and activity-based probes have elucidated the diversity and regulation of microbial bile acid-transforming enzymes, highlighting the complexity of the gut microbiome-bile acid-host interaction (22, 30).

### 2.2 Impact of microbial diversity on bile acid pool composition

Gut microbial diversity is a principal determinant of the composition and proportion of bile acids (BAs) in the intestinal pool and hence of their signaling properties. Primary bile acids secreted into the intestine undergo deconjugation, oxidation, epimerization, and 7α-dehydroxylation, generating a diverse array of secondary bile acids that modulate host metabolism and immune homeostasis (31–33). Microbial enzymes such as bile salt hydrolase (BSH) and bile acid:Na+ symporter family proteins mediate deconjugation and conjugation with alternative amino acids, expanding bile acid chemical diversity and altering their antimicrobial properties and receptor activation (34, 35). Variations in community composition therefore directly affect the abundance of bile acid-transforming taxa such as Lachnospiraceae and Bacteroidetes (36, 37).

Host factors including sex, diet, and disease state shape microbial diversity and, in turn, bile acid profiles. Sex-specific microbiota differences yield distinct bile acid pools, with higher oxidized bile acids in males and more conjugated primary bile acids in females (38, 39). Dietary interventions such as yeast β-glucan or black tea supplementation remodel microbial communities and bile acid metabolism, improving metabolic outcomes (40, 41). Conversely, dysbiosis with reduced diversity and loss of bile acid-transforming bacteria produces elevated primary and conjugated bile acids with diminished secondary bile acids, as observed in inflammatory bowel disease (IBD), metabolic syndrome, and cancer cachexia (8, 42, 43).

Mechanistically, dysbiosis impairs BSH and 7α-dehydroxylase activities, so conjugated bile acids accumulate while secondary bile acids decline, altering receptor activation and immune regulation (44, 45). Reduced signaling through farnesoid X receptor (FXR) and G protein-coupled bile acid receptor 1 (TGR5) compromises intestinal barrier integrity and immune tolerance, contributing to inflammatory and metabolic pathology (46, 47). Restoring microbial diversity through fecal microbiota transplantation, probiotics, or defined consortia producing anti-inflammatory secondary bile acids such as ursodeoxycholic acid and lithocholic acid has shown therapeutic potential in models of colitis and metabolic disease (48, 49).

Moreover, specific microbial taxa with bile acid-transforming capabilities, such as *Clostridium scindens*, *Blautia*, and *Bacteroides* species, are positively correlated with the abundance of unconjugated and secondary bile acids, reinforcing the link between microbial diversity and bile acid pool composition (50). Loss of these bacteria due to antibiotic treatment or disease leads to reduced bile salt hydrolase activity and altered bile acid profiles, exacerbating disease severity (51, 52). The microbial modulation of bile acid pools also influences colonization resistance against pathogens such as *Clostridioides difficile*, highlighting the role of microbial diversity in maintaining gut homeostasis (53).

Collectively, microbial diversity determines bile acid species composition and downstream receptor signaling; its disruption drives immune imbalance, and its restoration represents a plausible therapeutic strategy.

### 2.3 Bile acids’ feedback regulation on gut microbiota

Bile acids (BAs) exert marked antimicrobial activity in the intestinal lumen, shaping the composition, structure, and function of the gut microbiota. This activity reflects their detergent-like properties, which disrupt bacterial membranes and interfere with microbial metabolism. The effect is selective and dynamic, as different bile acid species inhibit or promote distinct taxa, generating a selective pressure that maintains a dynamic equilibrium between host-derived bile acids and microbial populations.

This reciprocity is exemplified by bile acids that suppress bile salt hydrolase (BSH)-producing bacteria such as *Lactobacillus murinus*, *Lactobacillus johnsonii*, and *Enterococcus faecalis*, as shown for Sanye Tablet (SYT) treatment in high-fat diet-induced hepatic steatosis (54). The resulting shift raises taurine-conjugated bile acids and modulates farnesoid X receptor (FXR)/fibroblast growth factor 15 (FGF15)/fibroblast growth factor receptor 4 (FGFR4) signaling, promoting hepatic bile acid synthesis and reducing lipid accumulation. L-theanine similarly decreases BSH-active *Lactobacillus* and *Bacteroides*, altering bile acid profiles and inhibiting intestinal FXR-FGF15 signaling (55).

Distinct bile acid species exert differential effects on microbial taxa. Secondary bile acids such as deoxycholic acid (DCA) and lithocholic acid (LCA), generated by bacterial 7α-dehydroxylation, can selectively promote or inhibit growth; arabinoxylan supplementation in obese mice enriched taxa with enhanced 7α-dehydroxylation activity, elevating secondary bile acid production and conferring metabolic benefit (56). Conversely, dysbiosis in inflammatory bowel disease (IBD) with reduced BSH activity yields increased primary and conjugated bile acids that reflect impaired microbial transformation (57).

The antimicrobial activity of bile acids is also implicated in disease contexts such as colorectal cancer liver metastasis (CRLM); in a murine model of CRLM, a deficiency in BSH-producing bacteria led to elevated conjugated primary bile acids that promoted neutrophil recruitment and tumor progression, and restoration of unconjugated bile acids counteracted these effects (58). Whether the same relationship holds in human CRLM has not yet been established, and this finding should be regarded as mechanistic and hypothesis-generating rather than clinically confirmed.

Mechanistically, bile acids influence microbial communities through receptor-mediated signaling pathways, including FXR and Takeda G protein-coupled receptor 5 (TGR5). For instance, gut microbiota-derived bile acids such as ω-muricholic acid (ωMCA) and hyocholic acid (HCA) stimulate GLP-1 secretion via TGR5, linking microbial bile acid metabolism to host metabolic regulation (59). Furthermore, the modulation of bile acid profiles by gut microbiota affects the expression of microbial bile acid metabolic enzymes, such as bile salt hydrolases, which exhibit substrate specificity and contribute to the diversity of the bile acid pool (60, 61).

Environmental factors and therapeutic interventions also modulate this bile acid-microbiota interplay. Chronic cold exposure alters gut microbiota composition and reduces microbiota-derived bile acids, exacerbating rheumatoid arthritis via TGR5-cAMP-PKA axis and inflammasome activation; supplementation with specific bile acids mitigates disease severity (62). Traditional Chinese medicines like Fuzi and Yixintai have been shown to remodel gut microbiota and bile acid metabolism, activating TGR5 signaling to reduce inflammation and improve metabolic outcomes in rheumatoid arthritis and chronic heart failure, respectively (63, 64). Dietary components such as capsaicin and inulin also influence gut microbial composition and bile acid metabolism, leading to improved lipid profiles and reduced inflammation (65, 66).

In summary, bile acids act as selective antimicrobial and signaling molecules that shape microbial ecology, while the microbiota reciprocally determines the composition of the bile acid pool. Disruption of this equilibrium contributes to diverse pathologies, and its targeted restoration represents a promising therapeutic avenue (**Figure 1**).

**Figure 1:**
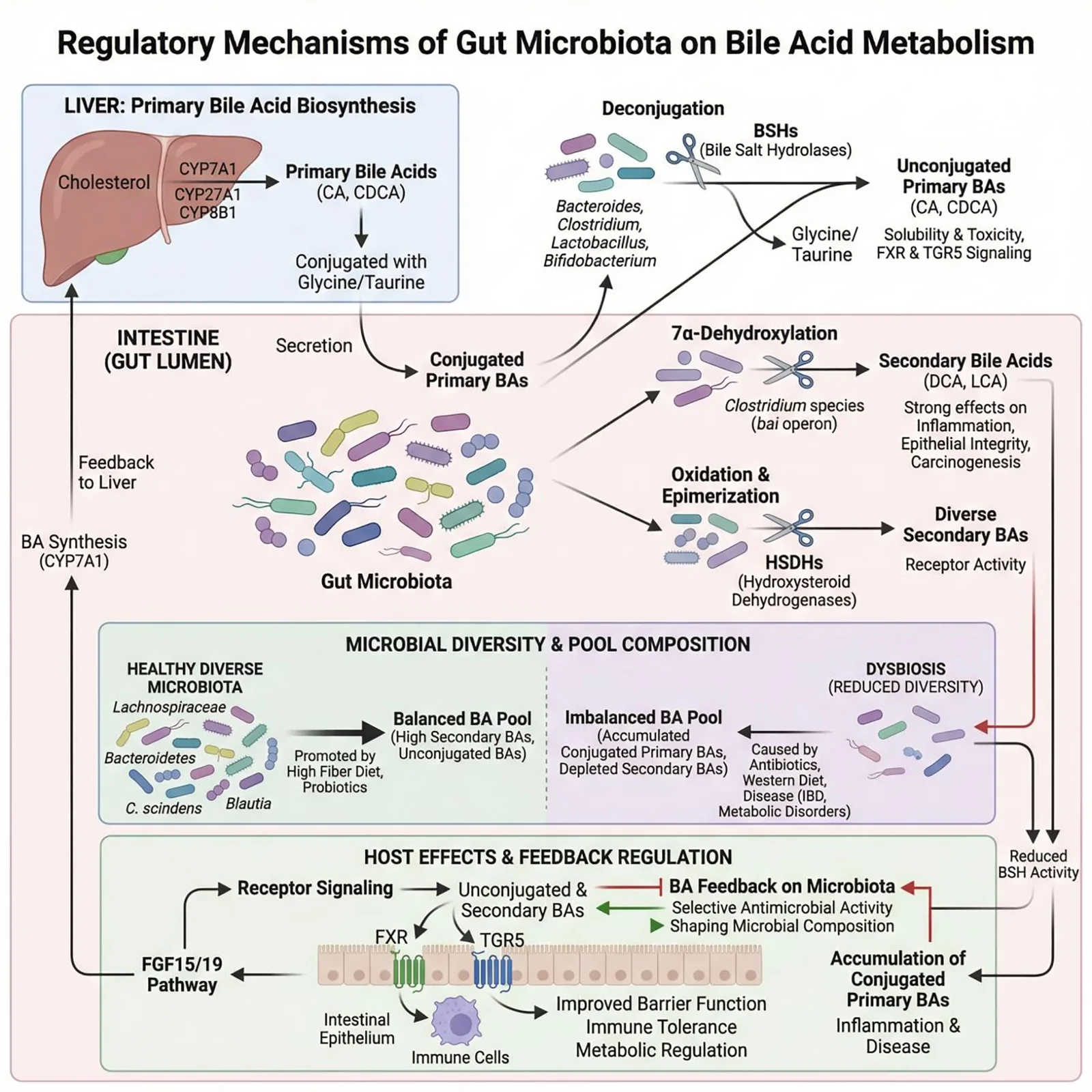
Bidirectional regulation between gut microbiota and bile acid metabolism. Schematic overview illustrating the bidirectional interactions between gut microbiota and bile acid (BA) metabolism. Primary bile acids synthesized from cholesterol in the liver are conjugated and secreted into the intestine, where gut microbiota catalyze deconjugation, 7α-dehydroxylation, oxidation, and epimerization through bile salt hydrolases (BSHs), bile acid–inducible (bai) operons, and hydroxysteroid dehydrogenases (HSDHs), generating a diverse pool of secondary bile acids. In turn, bile acids exert selective antimicrobial pressure and shape microbial community structure, favoring bile-tolerant or BA-transforming taxa while suppressing sensitive species. Dysbiosis disrupts this dynamic equilibrium, leading to altered bile acid composition, impaired receptor signaling, and immune imbalance.

## 3 Role of bile acid receptors and their signaling pathways in immune regulation

### 3.1 Farnesoid X receptor

Farnesoid X receptor (FXR) is a nuclear receptor predominantly expressed in liver and intestine that governs bile acid (BA) homeostasis by feedback inhibition of BA synthesis. In hepatocytes, FXR activation induces small heterodimer partner (SHP) and suppresses cholesterol 7α-hydroxylase (CYP7A1), the rate-limiting enzyme of BA synthesis, thereby preventing BA toxicity (67, 68). Intestinal FXR induces fibroblast growth factor 19 (FGF19; FGF15 in rodents), which reaches the liver through the portal circulation to inhibit BA synthesis, constituting a gut-liver endocrine axis (69, 70). This FXR-FGF19 axis additionally regulates glucose and lipid homeostasis, extending FXR function well beyond BA control (71, 72).

In addition to metabolic regulation, FXR activation exerts anti-inflammatory effects and maintains intestinal barrier integrity. FXR agonism suppresses pro-inflammatory cytokine expression and modulates immune cell function, including macrophage polarization and T cell responses, thereby protecting against intestinal inflammation and associated pathologies such as inflammatory bowel disease (IBD) and colitis-associated colorectal cancer (CAC) (73, 74). FXR deficiency or impaired signaling is associated with compromised intestinal barrier function, increased gut permeability, and dysregulated immune responses, which exacerbate inflammation and tissue injury (75, 76). Furthermore, FXR activation in intestinal stem cells promotes differentiation towards enteroendocrine cells, enhancing gut hormone secretion and contributing to metabolic benefits (77).

FXR-mediated FGF19 signaling also influences systemic energy metabolism by modulating skeletal muscle mass and function. Ileal FXR-FGF15/19 signaling promotes skeletal muscle protein synthesis via downstream pathways such as ERK1/2, indicating a gut-muscle axis regulated by FXR (72, 78). This axis may have therapeutic potential in conditions characterized by muscle atrophy, such as sarcopenia.

The gut microbiota profoundly influences FXR activity by modulating the BA pool composition. Microbial enzymes, such as bile salt hydrolases (BSHs), deconjugate and transform primary BAs into secondary BAs, which differentially activate or inhibit FXR. Dysbiosis alters BA profiles, impacting FXR signaling and contributing to metabolic and inflammatory diseases (71, 79). For example, probiotic administration or dietary supplements can modulate gut microbiota and BA metabolism, thereby influencing FXR-FGF19 signaling and ameliorating diseases like non-alcoholic fatty liver disease (NAFLD) and alcoholic liver disease (ALD) (80, 81). Conversely, environmental toxins or drugs disrupting gut microbiota or BA metabolism can impair FXR signaling, leading to liver injury and metabolic dysfunction (82, 83).

Pharmacological activation of FXR has shown promise in treating metabolic and liver diseases; however, systemic FXR agonists may cause side effects such as pruritus and dyslipidemia, prompting interest in tissue-specific or intestine-restricted FXR modulators (84, 85). Structural studies reveal that FXR functions as a heterodimer with retinoid X receptor (RXR), binding to specific DNA response elements to regulate gene transcription. The dynamic interplay between FXR, RXR, coactivators, and corepressors determines transcriptional outcomes, and novel FXR modulators targeting these interactions are under development (86–88).

In summary, FXR functions as a master regulator of bile acid homeostasis and a key modulator of metabolic, inflammatory, and immune processes in the liver and intestine, offering therapeutic potential across metabolic, inflammatory, and neoplastic diseases (89–91).

### 3.2 G protein-coupled bile acid receptor 1

The G protein-coupled bile acid receptor 1 (GPBAR1), also known as Takeda G protein-coupled receptor 5 (TGR5), is a membrane receptor expressed in intestinal enteroendocrine L-cells, brown and white adipose tissue, skeletal muscle, macrophages, liver, spleen, and pancreas, a distribution that underlies its integrative role in glucose and lipid metabolism and in immune modulation. Activation by bile acids or synthetic agonists stimulates glucagon-like peptide-1 (GLP-1) secretion, enhancing insulin release and glucose homeostasis; non-bile acid dual FXR/TGR5 agonists raise plasma GLP-1 and lower blood glucose in animal models (92, 93). TGR5 activation also induces thermogenic gene expression in brown adipose tissue and skeletal muscle, increasing energy expenditure; in diet-induced obese mice, ginsenoside Ro activated TGR5 to reduce obesity and insulin resistance by promoting GLP-1 secretion and enhancing energy expenditure (94).

Beyond metabolism, TGR5 restrains inflammation by suppressing NF-κB signaling in macrophages and other immune cells. In murine macrophages, sodium butyrate directed polarization toward an anti-inflammatory M2 phenotype via TGR5/β-arrestin2 signaling (95), and TGR5 activation attenuates liver inflammation and fibrosis in murine models of cholestasis and nonalcoholic fatty liver disease (96, 97). Its role extends to antiviral immunity, as gpbar1 activation induced interferon-stimulated genes in zebrafish (98), and to tissue regeneration, with lithocholic acid promoting skeletal muscle regeneration through TGR5-mediated AKT/mTOR signaling in mice (99).

Despite these promising preclinical findings, the development of TGR5 agonists faces challenges such as systemic side effects, including gallbladder filling, which has prompted the design of soft drugs with rapid metabolism to minimize adverse effects (100). Recent advances in structural biology, including cryo-electron microscopy and hydrogen/deuterium exchange mass spectrometry, have elucidated molecular determinants of TGR5-G protein interactions, facilitating rational drug design (101). Dietary bioactive compounds have also emerged as natural TGR5 activators with potential for managing diabetes and its complications through modulation of mitochondrial function and gut microbiota (102). TGR5 therefore represents a promising, though not yet clinically validated, therapeutic target linking bile acid signaling to metabolic and immune regulation (103, 104).

### 3.3 Other receptors: pregnane X receptor, vitamin D receptor, sphingosine-1-phosphate receptor 2

Pregnane X receptor (PXR), vitamin D receptor (VDR), and sphingosine-1-phosphate receptor 2 (S1PR2) are less extensively characterized bile acid (BA) receptors that nonetheless contribute substantially to BA detoxification, immune modulation, and inflammation within the gut-liver-immune axis. PXR is a promiscuous ligand-activated nuclear receptor that couples xenobiotic and BA detoxification to cholesterol homeostasis and immune tolerance. VDR, classically a mediator of vitamin D action, also binds secondary bile acids such as lithocholic acid (LCA) and thereby shapes immune responses and intestinal barrier integrity. S1PR2, a G protein-coupled receptor activated by conjugated bile acids, drives inflammatory signaling and cell migration in cholestatic liver injury and intestinal inflammation.

#### 3.3.1 PXR regulation of bile acid detoxification and immune tolerance

PXR (NR1I2) is highly expressed in liver and intestine, where it acts as a master regulator of xenobiotic and endobiotic metabolism. Activation by ligands including deoxycholic acid (DCA) and lithocholic acid (LCA) induces drug-metabolizing enzymes (e.g., CYP3A4) and transporters (e.g., MRP2, MDR1) that mediate bile acid detoxification and efflux; PXR also modulates cholesterol synthesis via HMGCR and PCSK9 and cooperates with FXR and LXR to maintain sterol and bile acid homeostasis (105, 106). Gut microbial metabolites regulate PXR as direct ligands or epigenetic modulators; short-chain fatty acids (SCFAs), for instance, enhance PXR expression by inhibiting histone deacetylases (106). Pharmacological activation by rifampicin and herbal products underscores its relevance to drug metabolism and to metabolic liabilities in polypharmacy (105).

Importantly, PXR contributes to immune tolerance by inhibiting NF-κB-mediated inflammatory pathways, thus attenuating intestinal and hepatic inflammation. In inflammatory bowel disease (IBD) and cholestatic liver diseases, diminished PXR activity correlates with increased inflammation and disrupted bile acid metabolism (107, 108). Experimental models demonstrate that PXR activation can alleviate inflammatory responses and restore bile acid homeostasis, highlighting its therapeutic potential. Moreover, PXR modulates the expression of transporters and enzymes critical for bile acid circulation and detoxification, as evidenced by studies showing that PXR deficiency exacerbates cholestatic injury and that PXR agonists can reverse such effects (109, 110). PXR also mediates crosstalk between the gut microbiota and host immunity, influencing drug metabolism and inflammatory responses (111, 112).

#### 3.3.2 VDR in immune regulation and intestinal barrier function

The vitamin D receptor (VDR) is a nuclear receptor activated by the active form of vitamin D (1,25-dihydroxyvitamin D3) and certain secondary bile acids, notably lithocholic acid (LCA). VDR is expressed in various immune cells and intestinal epithelial cells, where it modulates immune responses, maintains epithelial barrier integrity, and regulates antimicrobial peptide expression (113, 114). VDR activation suppresses pro-inflammatory cytokine production and promotes regulatory T cell differentiation, contributing to immune homeostasis in the gut and other tissues (115, 116). In murine models, VDR deficiency leads to altered bile acid composition and impaired bile acid excretion, underscoring its role in bile acid metabolism (114). Moreover, VDR signaling mitigates intestinal inflammation, as demonstrated by the attenuation of colitis severity upon LCA administration in a VDR-dependent manner (117).

VDR also influences hematopoiesis by mediating the effects of secondary bile acids on myeloid progenitor expansion, linking microbiota-derived metabolites to systemic immune regulation (118). Clinical studies associate vitamin D deficiency and VDR polymorphisms with inflammatory and autoimmune diseases, including IBD and liver disorders, suggesting that VDR modulation may have therapeutic benefits (113, 115). In the context of the gut microbiome, VDR expression is modulated by microbial metabolites and, reciprocally, VDR signaling shapes microbial composition and function (116, 119). Probiotic administration has been shown to upregulate colonic VDR expression, further linking microbiota and vitamin D pathways in mucosal immunity (116).

#### 3.3.3 S1PR2 in inflammatory signaling and cell migration

Sphingosine-1-phosphate receptor 2 (S1PR2) is a G protein-coupled receptor activated by conjugated bile acids, such as taurocholic acid (TCA), and the lipid mediator sphingosine-1-phosphate (S1P). S1PR2 is expressed in hepatocytes, macrophages, and cholangiocytes, where it regulates inflammatory signaling pathways, cytoskeletal dynamics, and cell migration (108, 120). Activation of S1PR2 promotes hepatic stellate cell activation via the p38 MAPK/YAP pathway, contributing to liver fibrosis under cholestatic conditions (121). In macrophages, S1PR2 signaling modulates pro-inflammatory cytokine production and recruitment to sites of liver injury, influencing the progression of vanishing bile duct syndrome and other cholestatic diseases (120).

S1PR2 also mediates bile acid-induced modulation of smooth muscle contractility in the intestine and impacts bile acid transport and synthesis through crosstalk with other receptors and signaling cascades (122, 123). Pharmacological inhibition of S1PR2 attenuates cholestatic liver injury, inflammation, and fibrosis, highlighting its potential as a therapeutic target (124, 125). Furthermore, S1PR2 is implicated in the progression of pancreatic cancer metastasis and non-alcoholic fatty liver disease-related hepatocellular carcinoma via activation of oncogenic pathways such as PI3K/AKT/mTOR (126, 127). In the context of viral infections, S1PR2 facilitates bile acid-dependent human norovirus entry and replication in intestinal epithelial cells, suggesting a broader role in host-pathogen interactions (128).

Collectively, PXR, VDR, and S1PR2 integrate metabolic, detoxification, and immune regulatory functions within the gut microbiota-bile acid-immune axis, and their dysregulation contributes to inflammatory bowel disease, cholestasis, metabolic disorders, and cancer (**Figure 2**).

**Figure 2:**
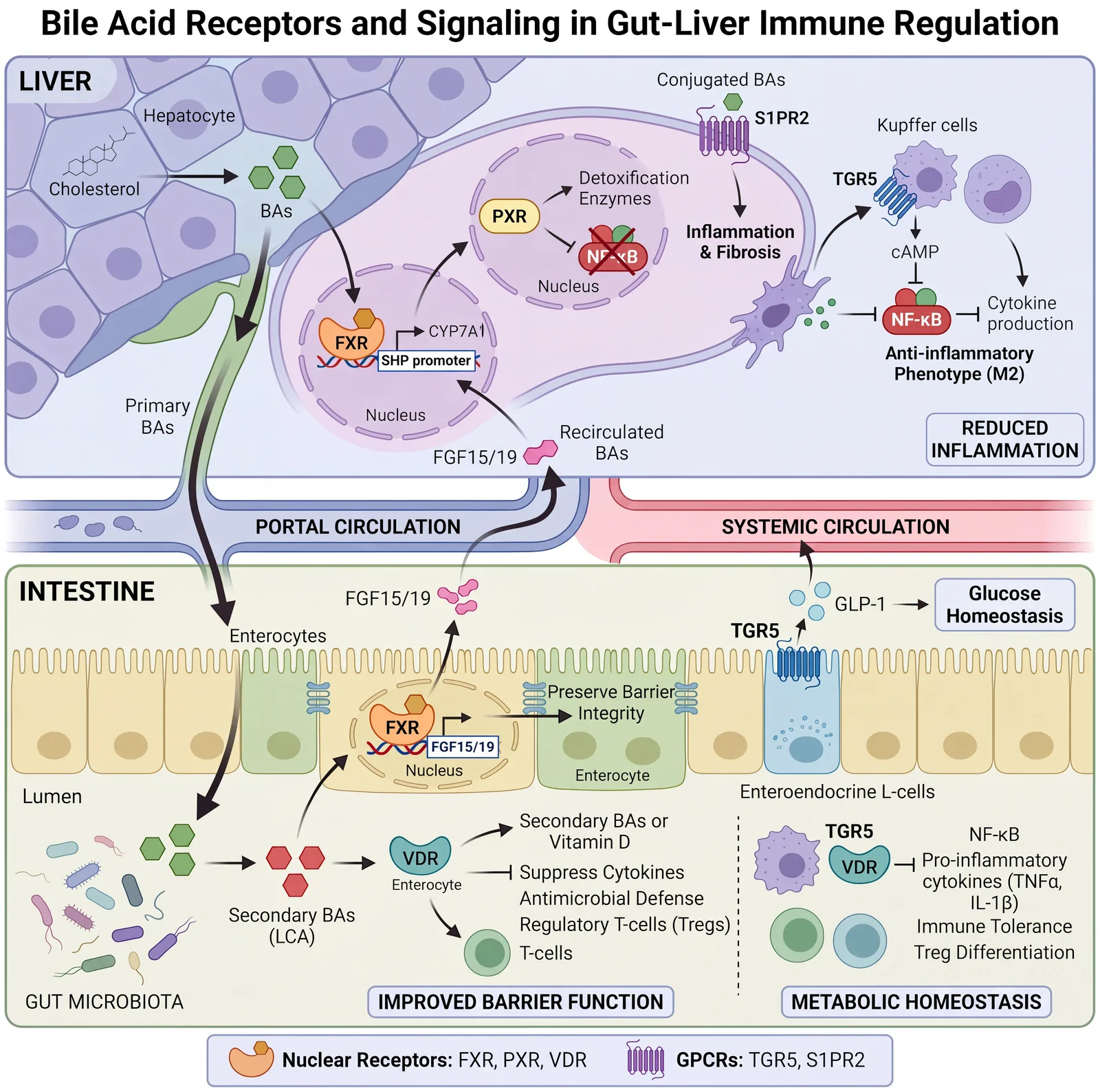
Bile acid receptor signaling pathways in immune and metabolic regulation. Integrated representation of bile acid receptor–mediated signaling pathways involved in immune modulation and metabolic regulation. Nuclear receptors, including farnesoid X receptor (FXR), pregnane X receptor (PXR), and vitamin D receptor (VDR), coordinate bile acid synthesis, detoxification, intestinal barrier integrity, and immune tolerance. Membrane-bound receptors, such as G protein-coupled bile acid receptor 1 (GPBAR1/TGR5) and sphingosine-1-phosphate receptor 2 (S1PR2), regulate inflammatory signaling, glucose metabolism, energy expenditure, and immune cell polarization through downstream pathways including NF-κB, MAPK, cAMP-PKA, AKT-mTOR, and Hippo-YAP signaling. Differential activation of these receptors by distinct bile acid species integrates microbial metabolism with host immune and metabolic homeostasis.

## 4 Pathological mechanisms of the gut microbiota-bile acid-immune axis in diseases

The immunometabolic consequences of bile acid signaling are highly dependent on bile acid species–specific receptor activation patterns (**Table 1**), underscoring the functional heterogeneity of primary, secondary, and derivative bile acids.

**Table 1:** Bile acid species–receptor specificity and immune–metabolic functions.

Bile acid species	Primary/Secondary	Major microbial transformation	Dominant receptor(s)	Key downstream signaling pathways	Immune regulatory effects	Metabolic effects	Disease relevance
Cholic acid (CA)	Primary	Deconjugation	FXR	FXR–SHP–CYP7A1 suppression	Maintains immune tolerance; limits excessive inflammation	Regulates bile acid synthesis; lipid homeostasis	Cholestasis, NAFLD
Chenodeoxycholic acid (CDCA)	Primary	Deconjugation	FXR, VDR	FXR–FGF19; VDR-mediated transcription	Anti-inflammatory; epithelial barrier protection	Glucose and lipid regulation	IBD, metabolic syndrome
Deoxycholic acid (DCA)	Secondary	7α-dehydroxylation	TGR5, FXR, PXR	cAMP–PKA; NF-κB inhibition	Suppresses macrophage M1 polarization; limits Th17 responses	Enhances energy expenditure	NAFLD, colorectal cancer
Lithocholic acid (LCA)	Secondary	7α-dehydroxylation	TGR5, VDR, PXR	TGR5–cAMP; VDR signaling	Promotes Treg differentiation; inhibits Th17	Improves insulin sensitivity	IBD, MS, neuroinflammation
IsoLCA/3-oxoLCA	Secondary derivative	Oxidation/epimerization	RORγt (indirect)	RORγt antagonism	Direct suppression of Th17 differentiation	Minimal metabolic effect	IBD, autoimmune disease
Taurocholic acid (TCA)	Conjugated primary	Limited transformation	S1PR2	p38 MAPK–YAP	Promotes inflammatory macrophage recruitment	Cholestatic injury	PSC, biliary fibrosis
Ursodeoxycholic acid (UDCA)	Secondary (therapeutic)	Epimerization	FXR (weak), TGR5	Anti-apoptotic signaling	Immunomodulatory; cytoprotective	Improves bile flow	PBC, cholestasis
Hyodeoxycholic acid (HDCA)	Secondary	Microbial conversion	TGR5	GLP-1 secretion	Anti-inflammatory	Improves glucose metabolism	Metabolic syndrome

### 4.1 Inflammatory bowel disease

Inflammatory bowel disease (IBD), comprising Crohn’s disease and ulcerative colitis, is marked by chronic relapsing intestinal inflammation in which microbial dysbiosis and disordered bile acid (BA) metabolism are increasingly implicated. Patients show reduced microbial diversity, depletion of Firmicutes species involved in BA biotransformation, and enrichment of Proteobacteria. The resulting loss of bacterial deconjugation and 7α-dehydroxylation activity elevates luminal primary and conjugated bile acids while depleting secondary bile acids, disrupting bile acid pool homeostasis (8, 12, 57).

This altered profile has substantial consequences for mucosal immunity and barrier integrity, because bile acids signal through farnesoid X receptor (FXR) and G protein-coupled bile acid receptor 1 (TGR5) to restrain inflammation and preserve epithelial function. In IBD, both receptors are hypoactive owing to changes in bile acid composition and receptor expression; reduced FXR activation increases NF-κB signaling and inflammasome activation, aggravating mucosal damage. Experimentally, restoring FXR signaling through microbial modulation or receptor agonists ameliorates colitis by promoting mucosal repair and suppressing pro-inflammatory pathways (129–131), whereas TGR5 activation by lithocholic acid (LCA) inhibits macrophage M1 polarization and dampens inflammatory cytokine production (49, 132).

Moreover, the gut microbiota influences immune cell function through bile acid metabolites. Specific microbial-derived bile acid metabolites, including 3-oxolithocholic acid (3-oxoLCA) and isolithocholic acid (isoLCA), have been identified as modulators of T helper 17 (Th17) cell differentiation by inhibiting the key transcription factor RORγt, thereby attenuating pro-inflammatory Th17 responses implicated in IBD pathogenesis. Notably, the levels of these immunomodulatory bile acids and the bacterial genes responsible for their biosynthesis are significantly reduced in IBD patients, correlating inversely with Th17-associated gene expression and suggesting a direct link between microbial bile acid metabolism and immune dysregulation in IBD (133).

Therapeutic interventions targeting the gut microbiota-bile acid axis show promise in IBD management. Probiotics that enhance bile salt hydrolase activity and BA-inducible enzymes can restore bile acid transformation, rebalancing the bile acid pool and activating protective receptor signaling pathways. For example, *Panax notoginseng* saponins have been shown to modulate gut microbiota composition, increase conjugated bile acids such as taurocholic acid (TCA) and taurochenodeoxycholic acid (TCDCA), and restore the Th17/regulatory T cell (Treg) balance, resulting in amelioration of colitis in murine models (134). Additionally, fecal microbiota transplantation (FMT) and bile acid receptor agonists are being explored to re-establish microbial and bile acid homeostasis, thereby improving intestinal barrier function and immune regulation (130, 135).

In summary, dysbiosis-driven bile acid perturbation impairs FXR and TGR5 signaling in IBD, promoting intestinal inflammation, barrier dysfunction, and pathogenic Th17 responses; restoring this axis represents a promising therapeutic strategy.

### 4.2 Metabolic syndrome and nonalcoholic fatty liver disease

Metabolic syndrome (MetS) and nonalcoholic fatty liver disease (NAFLD) share pathophysiological mechanisms centered on bile acid receptor signaling. Dysregulated signaling through farnesoid X receptor (FXR) and Takeda G protein-coupled receptor 5 (TGR5) contributes to the insulin resistance and lipid abnormalities that characterize both conditions: FXR governs bile acid synthesis, lipid homeostasis, and glucose metabolism, whereas TGR5 modulates energy expenditure and inflammation. In NAFLD, impaired bile acid signaling promotes hepatic steatosis, inflammation, and progression to nonalcoholic steatohepatitis (NASH) and fibrosis (136–138).

The gut microbiota shapes this process by determining bile acid composition. In NAFLD, dysbiosis is associated with altered bile acid profiles, including increased deoxycholic acid (DCA), which aggravates liver injury and fibrosis through pro-inflammatory pathways and immune cell modulation (36, 139, 140). Dysbiosis additionally compromises intestinal barrier integrity, allowing translocation of bacterial products that fuel hepatic inflammation along the gut-liver axis (141, 142).

Interventions targeting bile acid signaling and the gut microbiota have improved metabolic and hepatic outcomes in NAFLD. FXR agonists such as obeticholic acid and dual FXR/TGR5 agonists improve lipid and glucose metabolism and reduce liver inflammation and fibrosis in clinical trials (143–145). Prebiotics, probiotics, and natural compounds including inulin, resveratrol, and baicalin remodel the microbiota and bile acid metabolism, enhancing insulin sensitivity and reducing hepatic lipid accumulation, largely in preclinical models (146–148). Bile acid metabolites such as hyodeoxycholic acid (HDCA) and ursodeoxycholic acid (UDCA) mediate several of these benefits (138, 149). The strength of evidence nevertheless ranges from randomized clinical trials to animal studies and should be weighted accordingly (150, 151).

### 4.3 Autoimmune diseases and neurodegenerative diseases

The gut microbiota modulates immune tolerance and neuroinflammation largely through bile acid metabolism. Bacterial genera including *Bacteroides*, *Clostridium*, and *Lactobacillus* convert hepatic primary bile acids into secondary species such as lithocholic acid (LCA) and deoxycholic acid (DCA), which signal through farnesoid X receptor (FXR) and G protein-coupled bile acid receptor 1 (GPBAR1) expressed on immune cells. Dysbiosis disrupts this conversion, impairs receptor activation, and thereby erodes immune tolerance, favoring autoimmune responses and chronic inflammation.

In autoimmune liver diseases such as primary biliary cholangitis (PBC) and autoimmune hepatitis (AIH), altered microbiota and bile acid profiles have been linked to immune-mediated bile duct injury and hepatic inflammation. Microbiota-derived bile acids also direct the balance between regulatory T cells (Tregs) and pro-inflammatory Th17 cells. In murine models, antibiotic-induced microbial shifts lowered DCA, suppressed FXR expression, and inhibited the NLRP3-IL17A axis, reducing Th17 differentiation and alleviating autoimmune inflammation. Together, these observations place the gut microbiota-bile acid-immune axis at the center of immune homeostasis.

Furthermore, bile acid signaling has been proposed to modulate neuroinflammation by influencing microglial activation and cytokine production within the central nervous system (CNS). Secondary bile acids and their derivatives have been reported to cross the blood-brain barrier in animal studies and to engage receptors on microglia and neurons, potentially regulating neuroinflammatory processes; however, direct evidence for physiologically relevant blood-brain barrier crossing and receptor engagement in humans remains limited, and this mechanism should currently be regarded as plausible rather than firmly established. Gut microbiota and bile acid signaling nonetheless constitute a key interface linking peripheral immune tolerance with neuroimmune regulation, highlighting their potential as therapeutic targets for autoimmune and neurodegenerative diseases.

Bile acid receptors have been implicated as candidate modulators in neurodegenerative diseases such as multiple sclerosis (MS), Parkinson’s disease (PD), and Alzheimer’s disease (AD), in which immune dysregulation and neuroinflammation are central. In MS, depletion of bacteria producing deoxycholic acid (DCA) and lithocholic acid (LCA) correlates with reduced intestinal levels of these immunoregulatory metabolites and an expanded pro-inflammatory Th17 compartment. In experimental autoimmune encephalomyelitis (EAE), a murine model of MS, DCA and LCA suppress Th17 differentiation and the effector phenotype of myelin-reactive T cells via FXR and GPBAR1, thereby ameliorating disease severity. This disease-modifying role has so far been demonstrated only in the murine model and remains to be confirmed in patients.

In Parkinson’s disease, alterations in gut microbiota composition and bile acid metabolism have been observed in patients prior to motor symptom onset, and dysbiosis is associated with elevated levels of toxic secondary bile acids and disrupted bile acid receptor signaling, which are hypothesized to contribute to neuroinflammation, α-synuclein aggregation, and dopaminergic neuronal loss. Therapeutic interventions targeting gut microbiota, including probiotics and fecal microbiota transplantation, have shown preliminary promise in restoring bile acid homeostasis and mitigating PD-like pathology in animal models and small, largely uncontrolled human case series; this evidence is still at an early, exploratory stage, and adequately powered controlled trials in PD patients are needed before conclusions can be drawn about clinical efficacy.

In Alzheimer’s disease, bile acid dysregulation is associated with neuroinflammation, blood-brain barrier disruption, and amyloid-beta accumulation, and bile acid receptors are hypothesized to affect neuronal energy metabolism, oxidative stress, and microglial activation. A single proteomic study reported that deoxycholic acid interacts with Nicastrin, a component of γ-secretase, and proposed that this interaction promotes amyloid-beta formation; this preliminary observation has not been independently replicated or shown to be causal and should be interpreted with caution. Sex-specific differences in bile acid profiles and receptor expression may further modulate susceptibility and progression. Overall, bile acid receptors emerge as candidate components of the microbiota-gut-brain axis in MS, PD, and AD, but the supporting evidence ranges from replicated murine data to isolated preliminary findings, and clinical validation remains a priority.

### 4.4 Hepatobiliary diseases and viral infections

Hepatobiliary disease and viral infection intersect through viral hijacking of bile acid metabolism and microbiota-dependent remodeling of the hepatic immune microenvironment. Hepatitis B virus (HBV) manipulates bile acid pathways to support replication and evade immune clearance: circular RNA hsa_circ_0004812, upregulated in chronic hepatitis B (CHB) and in HBV-infected hepatoma cells, impairs interferon-induced immune responses by sponging miR-1287-5p and regulating Follistatin-related protein 1 (FSTL1), thereby promoting viral persistence (152). HBV nonstructural protein NS5A additionally downregulates hepcidin (HAMP) through the MTF-1/MRE/hepcidin axis, perturbing iron homeostasis (153). Such strategies illustrate how hepatotropic viruses co-opt host metabolic and immune circuits centered on bile acid and iron metabolism.

The gut microbiota-bile acid axis is increasingly recognized as a critical regulator of the hepatic immune microenvironment, influencing the progression of liver diseases including viral hepatitis and hepatobiliary cancers. Dysbiosis and altered bile acid metabolism have been observed in chronic HBV infection, with reductions in beneficial short-chain fatty acid-producing bacteria and disruption of the intestinal microbiota-bile acid-host axis, which contribute to disease progression (154). In HBV-related hepatocellular carcinoma (HCC), integration of gut microbiome and liver tumor transcriptome analyses revealed that specific bacterial genera, such as *Bacteroides* and *Clostridium XIVa*, correlate with tumor immune microenvironment alterations mediated via serum bile acids, linking microbial metabolites to hepatic carcinogenesis (155). Similarly, the constitutive androstane receptor (CAR), a nuclear receptor activated by bile acids, is upregulated in cholestatic liver diseases and modulates drug metabolism genes, highlighting the metabolic-immune interface modulated by bile acids in hepatobiliary pathology (156).

Viral infections also modulate innate immune signaling pathways in the liver, with HBV triggering Toll-like receptor 2 (TLR2)-MyD88-IRAK4-Hippo signaling axis activation, leading to NF-κB and Hippo pathway crosstalk that regulates inflammatory responses. The Hippo pathway effector YAP directly controls IκBα expression, providing a negative feedback loop to NF-κB-mediated inflammation during HBV infection (157). Furthermore, chronic viral infections induce CD8+ T cell exhaustion characterized by altered intracellular calcium flux, reactive oxygen species production, and increased IL-10 secretion, impairing antiviral immunity and facilitating viral persistence (158). In hepatitis C virus (HCV) infection, the expansion of myeloid-derived suppressor cells (MDSCs) is driven by the lncRNA HOTAIRM1-HOXA1-miR124 axis, promoting immunosuppression and viral persistence (159). These immune regulatory mechanisms underscore the intricate viral modulation of hepatic immunity.

Beyond hepatitis viruses, other viral infections impact the hepatobiliary system. For example, rotavirus non-structural protein NSP1 enhances viral replication in hepatocytes, leading to biliary obstruction and immunopathology reminiscent of biliary atresia (160). Human polyomaviruses, including HPyV6 and HPyV7, have been detected in cholangiocarcinoma tissues and adjacent hepatocytes, suggesting a possible viral contribution to hepatobiliary carcinogenesis (161). Additionally, herpes simplex virus can cause disseminated infection with fulminant hepatitis even in immunocompetent hosts (162). The recent COVID-19 pandemic has also highlighted hepatobiliary involvement, with SARS-CoV-2 infection causing cholestasis, liver enzyme elevations, and potential triggering of autoimmune sclerosing cholangitis in pediatric patients (163, 164).

Therapeutically, antiviral agents such as glycyrrhetinic acid have demonstrated hepatoprotective effects by inhibiting high-mobility group box 1 (HMGB1)-mediated inflammatory signaling in viral hepatitis models (165). Immune checkpoint blockade targeting PD-L1 shows efficacy in restoring HBV-specific CD8+ T cell function in patients with less exhausted T cell phenotypes, though combination strategies may be required for those with higher exhaustion (166). Chimeric antigen receptor (CAR) T-cell therapies are emerging as promising modalities for treating hepatic viral infections and hepatobiliary tumors by engineering immune cells to target infected or malignant cells (167).

In summary, hepatobiliary diseases and viral infections are intricately linked through viral manipulation of bile acid metabolism and immune pathways, as well as modulation of the gut microbiota-bile acid axis that shapes the hepatic immune microenvironment (**Figure 3**).

**Figure 3:**
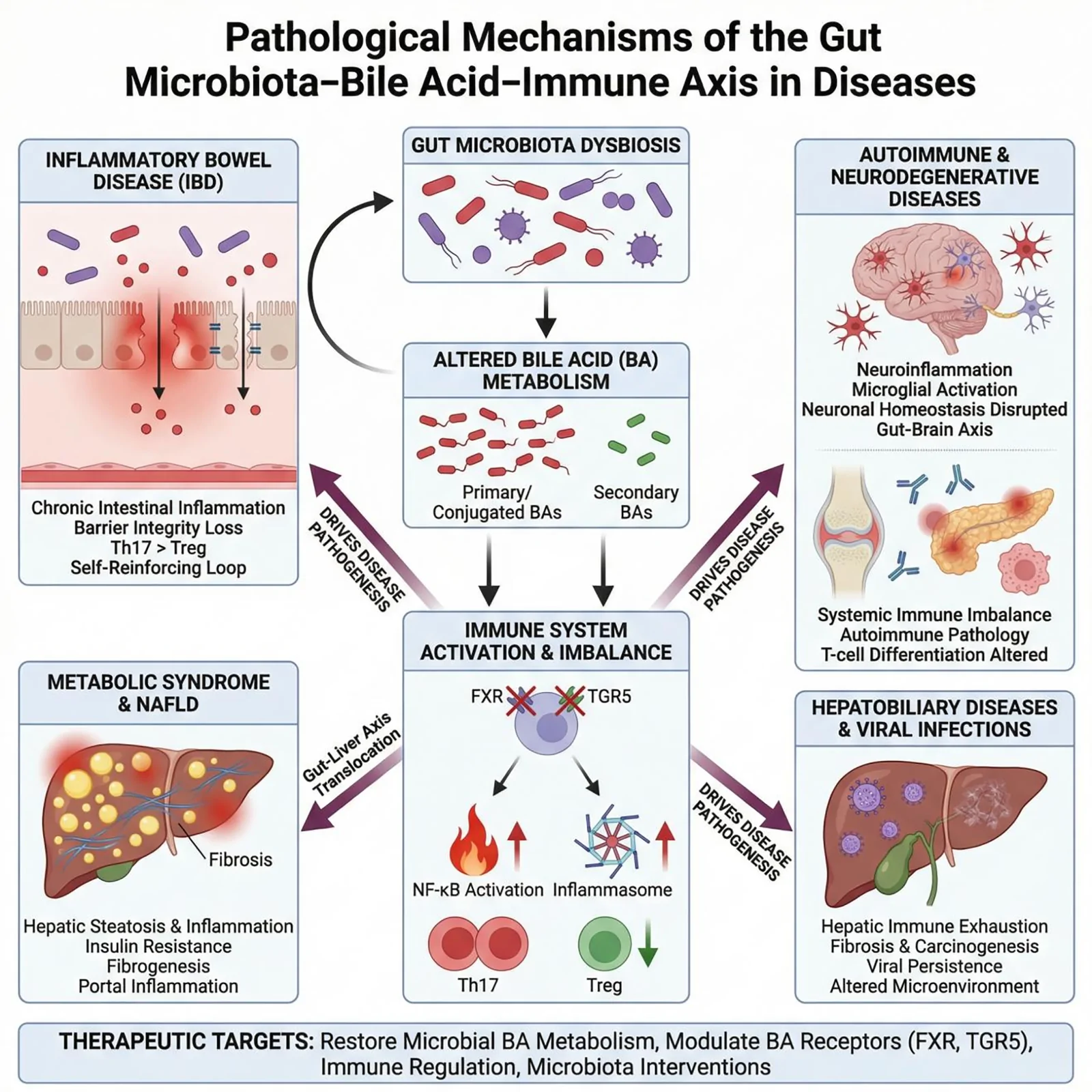
Pathological roles of the gut microbiota–bile acid–immune axis in human diseases. Pathophysiological mechanisms linking dysregulated gut microbiota–bile acid–immune interactions to disease development. Microbiota dysbiosis and altered bile acid pools impair bile acid receptor signaling, leading to chronic inflammation, metabolic dysfunction, and immune imbalance. In inflammatory bowel disease, reduced secondary bile acids and defective FXR/TGR5 signaling exacerbate mucosal inflammation and barrier disruption. In metabolic syndrome and nonalcoholic fatty liver disease, aberrant bile acid signaling promotes insulin resistance, hepatic steatosis, and fibrosis. In autoimmune and neurodegenerative diseases, bile acid–mediated immune dysregulation influences T cell differentiation, microglial activation, and neuroinflammation. In hepatobiliary diseases and viral infections, bile acid metabolism intersects with viral replication, immune exhaustion, and hepatic carcinogenesis.

## 5 Therapeutic transformation: strategies targeting the gut microbiota-bile acid-immune axis

### 5.1 Bile acid receptor agonists and antagonists

Bile acid receptors, chiefly the nuclear farnesoid X receptor (FXR) and the G protein-coupled bile acid receptor 1 (GPBAR1/TGR5), have become pivotal therapeutic targets because they integrate bile acid homeostasis, metabolism, and immunity. FXR agonists such as obeticholic acid (OCA) have been studied extensively in nonalcoholic steatohepatitis (NASH), where receptor activation improved liver histopathology and metabolic parameters by modulating bile acid synthesis and inflammatory pathways. Safety and efficacy concerns with selective FXR ligands have prompted development of dual FXR/GPBAR1 agonists and hybrid molecules addressing the multifactorial nature of metabolic liver disease (68, 168). Atorvastatin, for example, reset FXR and GPBAR1 signaling and protected against diet-induced steatohepatitis by improving bile acid profiles and gene expression patterns in adipose tissue and colon (144, 169).

TGR5 agonists have gained attention for their capacity to regulate metabolism and immune responses beyond hepatic effects. Activation of TGR5 enhances glucagon-like peptide-1 (GLP-1) secretion from enteroendocrine L cells, contributing to improved glucose homeostasis and insulin secretion. Experimental models demonstrated that TGR5 agonists alleviate neuropathic pain by inhibiting glial activation and neuroinflammation via potentiation of GABA_A receptor function in the spinal dorsal horn (170, 171). Furthermore, compounds such as notoginsenoside Ft1 act as TGR5 agonists while antagonizing FXR, thereby promoting fat browning, increasing adipose lipolysis, and improving insulin resistance in diet-induced obese mice, highlighting the therapeutic potential of dual modulation (172). The neurotrophic effects of bile acids mediated by TGR5 activation, including neurite outgrowth, further underscore the receptor’s broad physiological significance (173).

The therapeutic landscape also includes bile acid receptor antagonists designed to modulate overactive signaling pathways. FXR antagonists, such as FLG249 and novel 1,2,4-oxadiazole derivatives, have been characterized to inhibit FXR activity by disrupting coactivator and corepressor interactions, offering alternative strategies to regulate bile acid metabolism and glucose homeostasis (174, 175). Dual-function molecules combining FXR antagonism with GPBAR1 agonism, exemplified by (E)-7-ethylidene-lithocholic acid (7-ELCA) and 7β-isopropylchenodeoxycholic acid (7β-ipCDCA), have demonstrated efficacy in stimulating GLP-1 secretion and improving glucose metabolism in preclinical models, suggesting synergistic benefits of balanced receptor modulation (176, 177). Additionally, natural compounds such as sesamin and its metabolites exhibit FXR antagonistic activity, reducing gluconeogenesis-related gene expression and lowering blood glucose levels without adversely affecting bile acid metabolism, indicating potential for safe metabolic interventions (178).

In the context of cholestatic and inflammatory liver diseases, targeting bile acid receptors holds promise. FXR agonists like cilofexor and tropifexor are under clinical evaluation for primary sclerosing cholangitis (PSC), while combinatorial approaches activating GPBAR1 and inhibiting cysteinyl leukotriene receptor 1 (CYSLTR1) have shown efficacy in reversing drug-induced liver injury by modulating immune cell interactions (179, 180). Moreover, vincamine, a natural alkaloid, ameliorates hepatic fibrosis by activating the FXR-TGR5 pathway and inhibiting S100A4-mediated crosstalk between macrophages and hepatic stellate cells, highlighting the importance of bile acid receptor signaling in liver microenvironment regulation (181).

Overall, selective agonists, antagonists, and dual-acting hybrid molecules provide a versatile and evolving means of modulating metabolic, inflammatory, and immune pathways in NASH, metabolic syndrome, neuropathic pain, and cholestatic liver disease; optimizing efficacy and safety will require further definition of receptor-specific ligands, their molecular mechanisms, and their clinical outcomes.

### 5.2 Microbial regulation and probiotic intervention

Modulating the gut microbiota with probiotics, prebiotics, and microbial ecological agents has emerged as a means of restoring bile acid (BA) balance and thereby host immunity and metabolic health. Probiotics can enrich bacteria with bile salt hydrolase (BSH) activity that is essential for BA deconjugation and transformation. In inflammatory bowel disease (IBD), which is characterized by dysbiosis and aberrant BA metabolism, probiotic supplementation increased gut bacteria carrying BSH and BA-inducible enzymes, enhancing conversion of primary to secondary BAs and activating FXR and TGR5 to modulate intestinal immunity and inflammation (57).

Comparable effects have been reported for defined strains and consortia. *Lactobacillus fermentum* CKCC1858 alleviated hyperlipidemia in animal models by expanding short-chain fatty acid (SCFA)-producing bacteria and reshaping microbial metabolic function, indirectly influencing BA metabolism and lipid homeostasis (182). Multispecies probiotic complexes increased the abundance of *Lactobacillus* and *Bifidobacterium*, promoting BSH activity and 7α-dehydroxylation and raising secondary BAs such as deoxycholic acid and chenodeoxycholic acid (183).

Moreover, probiotics can improve intestinal barrier integrity and reduce systemic inflammation by modulating gut microbial metabolites, including BAs and SCFAs, which collectively contribute to restoring BA metabolic balance (184, 185). Clinical and animal studies indicate that probiotic intervention can suppress inflammatory cytokines and enhance immune homeostasis via BA receptor-mediated signaling pathways, though the magnitude of benefit and level of evidence differ considerably between human trials and preclinical models (186, 187). Prebiotics, non-digestible dietary fibers, serve as substrates for beneficial microbes, enhancing their growth and metabolic activity and thereby indirectly influencing BA metabolism and immune responses; the synergistic use of probiotics and prebiotics (synbiotics) may further potentiate these effects by fostering a favorable gut microbial environment conducive to balanced BA biotransformation (188, 189).

Microbial engineering now permits precise modulation of bile acid (BA) transformation. Engineered probiotics can express defined enzymes or bioactive molecules that act directly on BA metabolism and receptor signaling. A genetically modified strain expressing a high-affinity fragment of human PCSK9 nanobody lowered serum cholesterol and attenuated atherosclerosis in murine models by regulating LDL receptor expression and remodeling the gut microbiota, demonstrating that engineered microbes can influence lipid metabolism through gut-liver interactions (190). Probiotic spores encapsulated with prebiotics such as dextran ferment upon colonic colonization to generate anti-cancer SCFAs and reshape the microbiota in murine colon cancer models (191).

These engineered strains not only influence BA metabolism through enhanced enzymatic activities like bile salt hydrolase but also modulate host immune responses by altering microbial metabolite profiles, and synthetic biology tools allow precise control of microbial functions, including targeted BA deconjugation, 7α-dehydroxylation, and receptor agonism (192). Engineered probiotics can also be tailored to deliver therapeutic molecules locally within the gut, potentially reducing systemic side effects compared with conventional drugs, and the integration of omics technologies and computational modeling further facilitates the rational design of such strains (193). Despite these advances, all of the evidence described in this subsection derives from preclinical animal models; challenges remain in ensuring the safety, stability, and colonization efficiency of engineered probiotics in humans, and clinical translation has not yet been demonstrated.

### 5.3 Dietary and metabolic surgery interventions

Dietary composition strongly influences gut microbial structure and bile acid metabolism, with downstream effects on host metabolic and immune function. High-fiber, low-fat diets increase microbial diversity and enrich beneficial taxa such as *Akkermansia*, Lachnospiraceae, and Bifidobacteria that are associated with improved metabolic profiles (194, 195). Such diets enhance short-chain fatty acid (SCFA) production and regulate the bile acid (BA) pool by supporting primary BA synthesis and microbial conversion to secondary BAs; inulin supplementation, for example, increases taurine-conjugated bile acids and decreases fecal bile acid excretion, thereby restoring BA homeostasis (196, 197).

These shifts act through the farnesoid X receptor (FXR) and Takeda G protein-coupled receptor 5 (TGR5) to regulate lipid and glucose metabolism, energy expenditure, and immune responses (91, 198). Low-fat, high-fiber diets also reduce systemic inflammation and improve joint integrity by attenuating monocyte and macrophage activation, linking diet-induced microbiota changes to immune modulation (199). Collectively, these findings indicate that high-fiber, low-fat dietary patterns beneficially reshape the gut microbiota and bile acid pool and thereby improve metabolic and immunological health.

Metabolic surgeries such as Roux-en-Y gastric bypass (RYGB) and vertical sleeve gastrectomy (VSG) profoundly alter bile acid metabolism and gut microbial composition, contributing to benefits that exceed weight loss alone (200, 201). These procedures raise circulating bile acids, particularly secondary species, by enhancing microbial bile salt hydrolase (BSH) activity, thereby activating intestinal FXR-FGF19 signaling, improving glucose regulation, and reducing hepatic lipid accumulation (201, 202). Post-surgical enrichment of bile acid-metabolizing Clostridia and *Lactobacillus* species facilitates conversion of primary to secondary bile acids and further engages TGR5 and FXR (202, 203).

The resulting changes increase energy expenditure through brown adipose tissue thermogenesis and improve insulin sensitivity (204), while also modulating inflammatory signaling and intestinal barrier integrity (205, 206). Their causal contribution is supported by the observation that antibiotic depletion of the gut microbiota attenuates post-surgical metabolic improvement (204). Notably, pharmacological strategies such as ASBT inhibition combined with FGF15 activation reproduce part of this benefit, reinforcing bile acid signaling as the key axis through which metabolic surgery regulates energy homeostasis, glucose metabolism, and inflammation (207).

### 5.4 Emerging therapeutic strategies and future directions

Multi-omics integration—genomics, transcriptomics, proteomics, metabolomics, and microbiomics—has transformed the study of the gut microbiota-bile acid-immune axis and is driving the field toward precision medicine by profiling the interactions among host genetics, microbial communities, bile acid metabolism, and immune signaling. In hepatobiliary cancers such as hepatocellular carcinoma and cholangiocarcinoma, multi-omics analyses have identified microbial metabolite and bile acid signatures that track with tumor progression and therapeutic resistance and may serve as biomarkers for patient stratification (208, 209). In metabolic dysfunction-associated steatotic liver disease (MASLD), integrated omics have delineated dysbiosis-driven bile acid perturbation and immune dysregulation, guiding microbiota-targeted intervention (210, 211). In primary biliary cholangitis (PBC), such data have clarified the interplay of bile acid receptor signaling, microbiota alteration, and immune cell activation (9, 212), and comparable approaches in inflammatory bowel disease and neurodegenerative disorders have identified further molecular targets (8, 213). Realizing this potential will require standardized methodologies, longitudinal datasets, and robust computational tools for data interpretation.

Synthetic biology and artificial intelligence (AI) are reshaping therapeutic development for this axis. Synthetic biology permits engineering of microbial strains with customized metabolic capabilities, so that engineered probiotics and live biotherapeutics can restore microbial balance, generate beneficial bile acid derivatives, or deliver immunomodulatory molecules in IBD, metabolic disorders, and liver disease (214, 215). AI-driven computational modeling and machine learning analyze complex multi-omics datasets, predict microbial-host interactions, and identify optimal therapeutic targets, assisting the design of synthetic microbial consortia and of FXR or TGR5 modulators with improved specificity and fewer side effects (208, 216). AI-guided screening has accelerated discovery of FXR agonists such as nidufexor and isotschimgine for nonalcoholic steatohepatitis and cholestatic liver disease (217, 218), and AI-informed nanocarriers are being developed to deliver bile acid receptor modulators to hepatic tissue and enhance immunotherapeutic responses in liver cancer (219). Safety, regulatory approval, and clinical translation nevertheless remain unresolved and require sustained interdisciplinary collaboration.

Because diseases involving the gut microbiota-bile acid-immune axis are multifactorial, combination strategies targeting several pathways are increasingly favored over monotherapy, which is often limited by compensatory mechanisms and complex pathophysiology. In primary biliary cholangitis (PBC), regimens combining ursodeoxycholic acid (UDCA), farnesoid X receptor (FXR) agonists such as obeticholic acid, and peroxisome proliferator-activated receptor (PPAR) agonists achieve better biochemical response and symptom control than single agents (212, 220). In cholestatic liver disease and metabolic dysfunction-associated steatohepatitis, pairing bile acid receptor agonists with agents targeting inflammatory cascades, gut barrier integrity, and microbial dysbiosis has shown preclinical and clinical promise (221, 222). Extension to immuno-oncology—combining microbiota modulation, bile acid signaling manipulation, and immune checkpoint inhibitors—remains a hypothesis that has not been directly tested and should be regarded as such (208, 216). Adjunctive fecal microbiota transplantation, probiotics, and dietary interventions may further synergize with pharmacological agents (211, 223). Progress will depend on mechanistic understanding of pathway interactions, optimized dosing regimens, and biomarker-guided patient selection. Current and emerging therapeutic strategies targeting this axis are summarized in **Table 2**, together with their translational potential and unresolved challenges.

**Table 2:** Therapeutic strategies targeting the gut microbiota–bile acid–immune axis.

Therapeutic strategy	Target component	Mechanism of action	Representative examples	Target diseases	Advantages	Limitations/challenges
FXR agonists	FXR	Suppress bile acid synthesis; anti-inflammatory	Obeticholic acid, Cilofexor	NASH, PBC, PSC	Strong metabolic efficacy	Pruritus, dyslipidemia
Dual FXR/TGR5 agonists	FXR + TGR5	Enhance GLP-1; energy expenditure	Tropifexor, Nidufexor	Metabolic syndrome	Synergistic metabolic effects	Gallbladder effects
FXR antagonists	FXR	Reduce gluconeogenesis	Sesamin derivatives	Diabetes	Lower glucose without BA overload	Limited clinical data
TGR5 agonists	TGR5	GLP-1 secretion; anti-inflammatory	Natural bile acids, ginsenosides	Obesity, inflammation	Improves insulin sensitivity	Systemic side effects
Probiotics (BSH^+^)	Gut microbiota	Restore BA deconjugation	Lactobacillus, Bifidobacterium	IBD, NAFLD	Safe, low cost	Inter-individual variability
Engineered probiotics	Microbial metabolism	Precise BA transformation	Synthetic BSH strains	Metabolic disease	High specificity	Regulatory hurdles
Fecal microbiota transplantation	Microbial ecosystem	Restore microbial diversity	FMT	IBD, metabolic disease	Rapid microbiota reset	Safety, standardization
Dietary fiber intervention	Microbiota–BA pool	Promote SCFA & BA remodeling	Inulin, β-glucan	Metabolic syndrome	Non-invasive	Compliance issues
Metabolic surgery	BA circulation	Activate FXR–FGF19 axis	RYGB, VSG	Obesity, T2DM	Durable metabolic benefit	Invasive
AI-guided multi-omics therapy	Systems biology	Personalized targeting	Precision medicine platforms	Complex diseases	Individualized therapy	High cost

## 6 Research frontiers and challenges of the gut microbiota-bile acid-immune axis

### 6.1 Complexity and diversity in mechanistic studies

Mechanistic study of the gut microbiota-bile acid-immune axis is complicated by the diversity of bile acid species and of their receptors. Bile acids serve not only as digestive detergents but as signaling molecules acting through the farnesoid X receptor (FXR), G protein-coupled bile acid receptor 1 (GPBAR1/TGR5), pregnane X receptor (PXR), vitamin D receptor (VDR), and sphingosine-1-phosphate receptor 2 (S1PR2) (2). Each species differs in affinity and efficacy at these receptors, producing distinct downstream cascades that influence immune cell differentiation, cytokine production, and barrier integrity: primary bile acids predominantly activate FXR, suppressing bile acid synthesis and inflammation, whereas secondary species such as lithocholic acid derivatives preferentially engage GPBAR1 and modulate innate and adaptive immunity (23, 104). This ligand specificity generates a regulatory landscape that is highly sensitive to shifts in bile acid composition.

Compounding this complexity is the dynamic nature of the gut microbial community, which not only governs bile acid biotransformation but is itself shaped by bile acid-mediated antimicrobial activity and receptor signaling feedback loops. Dysbiosis, characterized by reduced microbial diversity and the expansion of bile-tolerant or pro-inflammatory taxa, alters the bile acid pool composition, often increasing the ratio of primary to secondary bile acids, as observed in inflammatory bowel disease (IBD) and cholestatic liver diseases (8, 221). Such alterations disrupt the activation patterns of bile acid receptors, leading to impaired immune regulation, increased intestinal permeability, and chronic inflammation. Moreover, individual variability in microbiota composition, influenced by genetics, diet, environmental exposures, and disease states, further modulates bile acid metabolism and receptor signaling outcomes, thereby affecting the reproducibility and interpretation of mechanistic studies (13, 224).

The complexity extends to the spatial and temporal dimensions of bile acid signaling. Bile acid receptors are differentially expressed along the gut-liver axis, including intestinal epithelial cells, immune cells within the lamina propria, hepatic parenchymal and non-parenchymal cells, and even extrahepatic tissues such as the kidney and brain (18, 225). This anatomical distribution enables bile acids to orchestrate localized and systemic immune responses, metabolic regulation, and barrier function. For instance, FXR activation in the intestine induces fibroblast growth factor 15/19 (FGF15/19) secretion, which acts on the liver to regulate bile acid synthesis and glucose metabolism, illustrating a tightly regulated enterohepatic feedback mechanism (226). Additionally, bile acid receptor signaling intersects with other metabolic and immune pathways, including inflammasome activation, nuclear factor-kappa B (NF-κB) signaling, and T cell differentiation via retinoid-related orphan receptor gamma t (RORγt), highlighting the pleiotropic effects of bile acids (104, 227).

Methodological challenges arise from this complexity and diversity. Variability in experimental models, including differences in microbiota composition across animal facilities, human cohort heterogeneity, and the use of *in vitro* systems lacking the full complement of microbial and host interactions, complicate the extrapolation of findings. Furthermore, bile acid pools are influenced by diet, circadian rhythms, and environmental chemicals, adding layers of regulatory nuance (2, 226). The dynamic and reciprocal interactions between microbiota and bile acids necessitate integrative multi-omics approaches combining metagenomics, metabolomics, transcriptomics, and immunophenotyping to unravel the mechanistic underpinnings effectively (14, 228).

In summary, mechanistic studies of the gut microbiota-bile acid-immune axis must contend with multiple interacting bile acid species and receptor pathways, individualized microbiota composition, and spatially distributed receptor expression, all of which complicate reproducibility; future research should emphasize personalized, systems-biology approaches to translate these mechanistic insights into clinical interventions.

### 6.2 Clinical translation barriers and standardization needs

The bidirectional and context-dependent behavior of bile acid receptors complicates the rational design of therapeutics targeting this axis. The farnesoid X receptor (FXR) and G protein-coupled bile acid receptor 1 (GPBAR1/TGR5) mediate divergent and sometimes opposing effects depending on ligand specificity, tissue distribution, and cellular context: FXR activation suppresses inflammatory pathways and improves metabolic homeostasis but may be deleterious in certain pathological settings, while systemic TGR5 activation can cause gallbladder filling or perturb glucose metabolism. Achieving therapeutic efficacy without off-target or paradoxical effects is therefore difficult.

This difficulty is compounded by the dependence of receptor signaling on gut microbiota composition and bile acid pool dynamics, which vary substantially between individuals and disease states. Tissue-specific or ligand-selective modulation, including biased agonists that preferentially activate beneficial pathways, offers a plausible solution but demands a deeper mechanistic understanding of receptor-ligand interactions and downstream effects. Without standardized biomarkers and robust models that capture this variability, translation of receptor-targeted interventions from bench to bedside will remain challenging (2, 17, 223, 228).

Microbial therapeutics—probiotics, engineered bacteria, and fecal microbiota transplantation (FMT)—offer a complementary route to modulating this axis, but their clinical translation is constrained by safety, stability, and standardization. Safety profiles remain incompletely characterized, particularly in immunocompromised patients and those with complex comorbidities, and risks including pathogen transmission, unintended immune activation, and horizontal transfer of antibiotic resistance genes demand stringent evaluation.

Maintaining viability, functional activity, and consistent composition of live biotherapeutics during manufacture, storage, and administration is likewise challenging, while donor variability and the absence of standardized screening, preparation, and delivery protocols for FMT produce inconsistent clinical outcomes and regulatory obstacles. Dosing regimens, treatment duration, and patient selection criteria also require standardization, and accurate prediction of efficacy and adverse events depends on mechanistic insight into the interactions between administered microbes, host bile acid metabolism, and immune responses. Regulatory frameworks must evolve accordingly to balance innovation with patient safety (8, 14, 223, 229, 230).

### 6.3 Application of multi-omics and systems biology

Multi-omics and systems biology have advanced understanding of the gut microbiota–bile acid–immune axis by enabling integrative analyses that dissect complex molecular interactions and regulatory networks. Combining genomics, metabolomics, and immunomics captures the dynamic interplay between microbial communities, their metabolic outputs, and host immune responses in ways single-omics studies cannot. Metagenomic and transcriptomic studies have identified microbial taxa and host gene expression changes involved in bile acid metabolism and immune modulation in inflammatory bowel disease, metabolic disorders, and infectious disease (231–233).

Metabolomic profiling quantifies bile acid species and other microbial metabolites that act as signaling molecules influencing immune cell differentiation, barrier function, and inflammatory pathways (234, 235), while immunomics data, including immune cell phenotyping and cytokine profiling, show how these metabolites regulate innate and adaptive immunity, for example by shifting the Treg/Th17 balance (236, 237). Integrative analyses have further clarified the molecular basis of disease resistance phenotypes and therapeutic responses in inflammatory bowel disease and sepsis, linking indole derivatives and bile acids to immune regulatory pathways and clinical outcomes (238, 239).

Systems biology frameworks build predictive models that integrate heterogeneous omics datasets to identify key regulatory nodes and pathways within the axis. Machine learning applied to such data predicts disease susceptibility and treatment response and supports patient stratification: in Crohn’s disease, microbiome-metabolome signatures involving bile acid metabolism predicted responsiveness to anti-TNF therapy and identified *Bifidobacterium* abundance as a mediator of bile acid-dependent immune regulation (240). Similar integration has yielded microbial and metabolic biomarkers correlating with vaccine efficacy, immunotherapy outcome, and metabolic disease progression (236, 241, 242), and has revealed transcription factors that interpret gut-liver crosstalk to regulate hepatic metabolism and inflammation (243). Computational tools such as weighted gene correlation network analysis (WGCNA) and constraint-based modeling extend this capacity to hypothesis testing and drug discovery (244, 245).

Despite these advances, challenges remain in standardizing multi-omics data integration, addressing individual heterogeneity, and translating findings into clinical practice. The dynamic and context-dependent nature of the gut microbiota and host immune responses necessitates longitudinal and spatially resolved multi-omics studies, as well as the incorporation of environmental and lifestyle factors. Emerging technologies such as single-cell omics, spatial metabolomics, and artificial intelligence-driven predictive modeling hold promise for overcoming these limitations and for enabling the design of personalized therapeutic strategies that modulate the gut microbiota–bile acid–immune axis effectively (235, 239, 246). In summary, the integration of multi-omics and systems biology provides a powerful platform to comprehensively elucidate the mechanisms underlying the gut microbiota–bile acid–immune axis and to develop predictive models that guide individualized treatment, ultimately advancing precision medicine in immune-mediated and metabolic diseases.

### 6.4 Emerging technologies driving theoretical and applied advances

Single-cell sequencing and spatial omics have transformed the resolution at which cell-specific signaling within this axis can be examined. Single-cell sequencing resolves heterogeneous populations into distinct transcriptional and metabolic states underlying responses to bile acids and microbial metabolites; in Alzheimer’s disease it has clarified gut-brain-immune interactions by identifying immune and epithelial subsets that modulate disease progression through receptor signaling (247), and in immune regulation it has delineated T cell exhaustion pathways and metabolic reprogramming (248). Spatial omics preserves tissue architecture, mapping cell-specific receptor expression and metabolite localization within gut and liver microenvironments—context that is essential for understanding how FXR and TGR5 orchestrate localized metabolic and immune responses (249, 250).

Artificial intelligence (AI) has emerged as a transformative tool to integrate and analyze the vast datasets generated by these high-dimensional technologies. Machine learning algorithms facilitate the identification of key regulatory nodes within receptor signaling networks and metabolic pathways, accelerating the discovery of novel drug targets. For example, AI-assisted analyses have been instrumental in predicting microbiota-metabolite interactions that influence systemic diseases such as metabolic syndrome and pulmonary hypertension, where gut microbial metabolites modulate host receptor signaling and immune responses (251, 252). Moreover, AI-driven models enable the stratification of patient populations based on microbiome and metabolomic profiles, supporting precision medicine approaches that tailor interventions targeting the gut microbiota-bile acid-immune axis. The integration of AI with organ-on-chip and *in vitro* gut models further enhances the capacity to simulate human-specific host-microbe interactions, thereby refining therapeutic development pipelines (253, 254).

Together, these emerging technologies deepen mechanistic insight into cell-specific receptor signaling and metabolic regulation within the gut microbiota-bile acid-immune axis, and they support the rational design of microbiome-targeted therapies, including probiotics, fecal microbiota transplantation, and small-molecule modulators of bile acid receptors, for metabolic, inflammatory, and neurodegenerative diseases linked to this axis (255, 256). Beyond bile acids, other microbiota-derived metabolites also shape the response to immunotherapy; for example, Lactobacillus salivarius-derived indole-3-lactic acid suppresses anti-PD-1 efficacy in esophageal squamous cell carcinoma by driving terminal exhaustion of CD8+ T cells (257).

## 7 Conclusion

The gut microbiota–bile acid–immune axis constitutes a pivotal nexus linking microbial metabolism to host immune and metabolic homeostasis. It has emerged as a determinant of the pathogenesis and progression of a wide spectrum of chronic diseases and thus as both a biomarker and a therapeutic target, with bile acid receptors transducing the diverse signals that fine-tune immune responses and metabolic balance.

Substantial progress has been made in defining bile acid receptor signaling and microbial modulation, yet integrating these findings into coherent clinical strategies remains difficult. Experimental work has established the bidirectional communication between gut microbes and bile acid metabolism, showing how microbial shifts reshape bile acid pools and receptor activation profiles and thereby immune cell function and systemic metabolism; clinical observations correspondingly link dysregulation of this axis to inflammatory bowel disease, metabolic syndrome, and liver disorders.

Therapeutic approaches now span microbial interventions, receptor-specific agonists and antagonists, and dietary modification, each with distinct advantages and limitations. Microbiota modulation through probiotics, prebiotics, or fecal microbiota transplantation seeks to restore beneficial communities and bile acid composition but is limited by variable patient response and uncertain long-term safety. Receptor-targeted therapies offer mechanistic specificity yet face off-target effects and receptor crosstalk, while dietary strategies are accessible and non-invasive but require personalization.

Advancing clinical translation will require bridging mechanistic insight with well-designed trials: identifying the patient subgroups most likely to benefit, developing robust biomarkers for monitoring therapeutic efficacy, and defining long-term outcomes through multi-omics technologies, computational modeling, and longitudinal cohort studies. With such integration, the gut microbiota–bile acid–immune axis offers a promising avenue for precision-targeted therapies against chronic diseases rooted in immune and metabolic dysregulation.
